# Retracted: Physical Activity: An Important Adaptative Mechanism for Body-Weight Control

**DOI:** 10.1155/2017/5876250

**Published:** 2017-07-02

**Authors:** International Scholarly Research Notices

International Scholarly Research Notices has retracted the article titled “Physical Activity: An Important Adaptative Mechanism for Body-Weight Control” [1]. The article was found to contain a substantial amount of material from the following published articles: “Tappy, L., Binnert, C. and Schneiter, P. (2003) ‘Energy expenditure, physical activity and body-weight control', Proceedings of the Nutrition Society, 62(3), pp. 663–666. doi: 10.1079/PNS2003280,” and “James A. Levine: Nonexercise activity thermogenesis (NEAT): environment and biology American Journal of Physiology - Endocrinology and Metabolism Published 1 May 2004 Vol. 286 no. 5, E675–E685 DOI: 10.1152/ajpendo.00562.2003.” The first author Carmine Finelli accepts responsibility for this and apologizes to Tappy et al. and Levine.
